# Internal Stress of Titanium-Based Nitride with Penetration Depth and Surface Roughness by sin^2^ψ Method Using HR-XRD

**DOI:** 10.3390/nano15110813

**Published:** 2025-05-28

**Authors:** Sungju Yoo, Eunpyo Hong, Youngkue Choi, Heesoo Lee

**Affiliations:** 1 School of Materials Science and Engineering, Pusan National University, Busan 46241, Republic of Korea; yoosj@pusan.ac.kr; 2 Mechanical & Material Technology Center, Korea Testing Laboratory, Jinju 52852, Republic of Korea; ephong@ktl.re.kr

**Keywords:** titanium-based nitride, internal stress, HR-XRD, coating thickness, surface roughness

## Abstract

The test method for internal stress of titanium-based nitride was optimized via penetration depth and surface roughness. Through the test method, the variations in the mechanical properties due to the ratio of the carbon gradient layer were investigated in terms of internal stress. TiN coatings were deposited on SUS 304 using RF/DC magnetron sputtering, and the penetration depth was adjusted by varying the X-ray power of HR-XRD for test specimens with the same coating thickness of 1 μm. The gradient of diagram for internal stress remained constant regardless of the penetration depth, and this was attributed to the analysis of internal stress focusing on the preferred growth orientation of the coating and excluding the influence of the substrate. In addition, we tested different surface roughness values (0.01 Sa, 0.02 Sa, and 0.03 Sa) to observe the effect on internal stress measurement. The results showed negligible difference in internal stress, confirming that this measurement method is valid for coatings with a surface roughness of 0.03 Sa or less. The test method was applied to analyze the carbon-doped TiZrN coating. TiZrN coatings were deposited on SUS 304, and coating thicknesses of 0.5 μm, 1 μm, and 2 μm were used to control the ratio of the carbon gradient layer. After applying the carbon paste for carbon doping, the TiZrN coating was irradiated with a pulsed laser. The compressive internal stress increased from −1263 MPa to −1687 MPa at a coating thickness of 0.5 μm, where the ratio of the carbon gradient layer was the highest. It was confirmed that the increase in internal stress with the ratio of the carbon gradient layer improved the mechanical properties of the carbon-doped TiZrN coating by laser carburization.

## 1. Introduction

Titanium-based nitride coatings, which are ceramic coatings formed by the combination of transition metals and non-metallic elements, have excellent thermal and mechanical properties, attracting attention in various industries, including aerospace. For applications in extreme environments such as aerospace, greater mechanical properties and durability are required [1,2,3]. Research has been actively conducted on doping with foreign elements to improve mechanical properties and control internal stress [4,5,6]. In particular, it is important to accurately evaluate internal stress, as excessive internal stress can lead to the delamination or cracking of the coating [7].

There are various methods available for evaluating internal stress depending on the material and purpose, and the sin^2^ψ method using HR-XRD, a non-destructive method, can be applied to ceramic coatings. The sin^2^ψ method quantifies internal stress by combining Bragg’s law, which is an X-ray-related law, and Hooke’s law, which is a stress-related law [8,9,10]. Furthermore, unlike conventional XRD, HR-XRD utilizes a parallel beam to align the X-rays incident on the specimen and a Chi-Phi-x-y-z 5-axis sample stage that allows specimen rotation. This enables internal stress calculation, even at low-angle peaks, which corresponds to the preferred growth orientation of the coating, thereby improving accuracy.

Laser carburization can be used to dope carbon into titanium-based nitride coatings, forming a carbon gradient layer within the coating through the diffusion of thermal energy. The carbon gradient layer refers to a layer in which the carbon concentration gradually changes within the coating. This method simplifies the process while simultaneously achieving excellent properties [11,12,13]. Carbon can stably occupy interstitial or substitutional positions in the TiN lattice, enhancing the mechanical properties of the coating through lattice distortion. It is crucial to investigate the difference in property improvements depending on the ratio of the carbon gradient layer within the coating [14,15].

In this study, TiN coatings were deposited on SUS 304, and the test method for internal stress was optimized using the sin^2^ψ method with HR-XRD. Then, this test method was applied to the carbon-doped TiZrN coatings, and the variations in the mechanical properties by carbon gradient layer were investigated in terms of internal stress. The cross-sectional microstructure of the coating was analyzed using SEM. Surface roughness was analyzed using a confocal microscope. Carbon concentrations according to coating depth were analyzed using ToF-SIMS. The crystal structure analysis resulting from carbon doping was conducted using HR-XRD, and the lattice constant variations were calculated using Rietveld refinement. The sin^2^ψ method was used to calculate the internal stress quantitatively, and HR-XRD and a Nanoindenter were used to obtain the variables required for calculation. Hardness was measured using a Nanoindenter to confirm the improvement in the mechanical properties of TiZrN coatings achieved by carbon doping.

## 2. Materials and Methods

TiN (Ti = 99.99 wt%) coatings were deposited onto SUS 304 substrates using RF/DC magnetron sputtering. Prior to deposition, the surfaces of SUS 304 were cleaned using ultrasonic waves and ethanol to remove contaminants and enhance adhesion. To investigate the variation in internal stress according to X-ray penetration depth in HR-XRD from the coating surface, TiN coatings were uniformly deposited with a thickness of 1 μm. Additionally, specimens with surface roughness values of 0.01 Sa, 0.02 Sa, and 0.03 Sa were prepared to evaluate the influence of surface roughness on internal stress and to determine an optimal range. Subsequently, TiZrN (Ti:Zr = 50:50 wt%) coatings were deposited using the same method. To control the ratio of the carbon gradient layer, the coating thickness was deposited to 0.5 μm, 1 μm, and 2 μm by controlling the deposition time. The deposition conditions are shown in Table 1.

To prepare the paste for carbon doping, graphite powder (20 μm) and polyvinylidene fluoride (PVDF) were mixed in a 9:1 ratio, and 1-Methyl-2-pyrrolidinone was used to control the viscosity. The carbon paste was applied to the TiZrN coating using screen printing and dried at 80 °C to improve adhesion. A Nd:YAG pulsed laser ablation system (LSX-213) was used for carbon doping through laser carburization. The laser was irradiated 10 times with a laser power of 50%, a wavelength of 213 nm, and a frequency of 10 Hz, utilizing a spot diameter of 200 μm and a distance between spots of 250 μm. After carburization, the remaining carbon paste on the surface was removed using ultrasonic waves and ethanol.

The microstructure of the coatings was analyzed using a Scanning Electron Microscope (SEM, MIRA3, TESCAN, Brno-Kohoutovice, Czech Republic). The surface roughness of the coatings was analyzed using a confocal microscope (Leica, DCM8, Wetzlar, Germany). Carbon concentrations according to coating depth were analyzed using ToF-SIMS (ToF-SIMS 5, ION-TOF GmbH, Münster, Germany). The crystal structure and internal stress of the coating were analyzed using high-resolution X-ray diffraction (HR-XRD, Smartlab, Rigaku, Tokyo, Japan) within the range of 20° to 100° (step scan: 0.02°/2θ). The wavelength of the X-ray was Cu Kα (1.5418 Å), and the X-ray power was set to 3 kW, 6 kW, and 9 kW to control the penetration depth. The lattice constant variation of the coatings was analyzed by Rietveld refinement with X’pert HighScore (Malvern Panalytical, Almelo, The Netherlands). The variations in internal stress were evaluated using the sin^2^ψ method with HR-XRD. The improvement in hardness due to carbon doping was measured using a Nanoindenter (Helmut Fischer, FISCHERSCOPE HM2000, Baden-Württemberg, Germany) under conditions of a maximum load of 50 mN, an applied load of 10 s, and a constant load.

## 3. Results and Discussion

**Figure 1:**
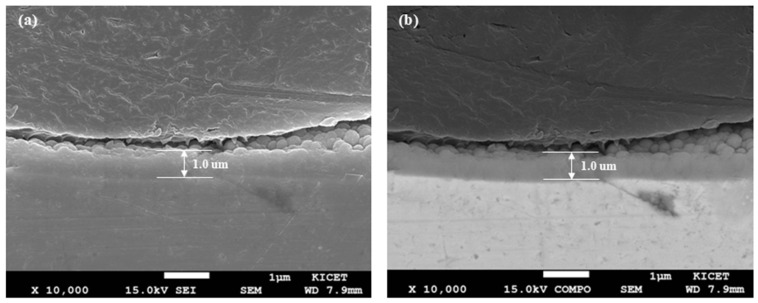
Cross-sectional SEM images of TiN coating: (**a**) SEI image; (**b**) COMPO image.

In Figure 1, the coating thickness was confirmed to be 1 μm through microstructure analysis to fix the variable of coating thickness before measuring internal stress using HR-XRD. Since variations in coating thickness can lead to changes in internal stress, making it difficult to accurately evaluate the measurement method, the X-ray power was adjusted to vary the analysis range. Tests were conducted on the same specimen by adjusting the X-ray power of HR-XRD to 3 kW, 6 kW, and 9 kW, focusing on the (111) plane, which is the preferred growth orientation of TiN.

**Figure 2:**
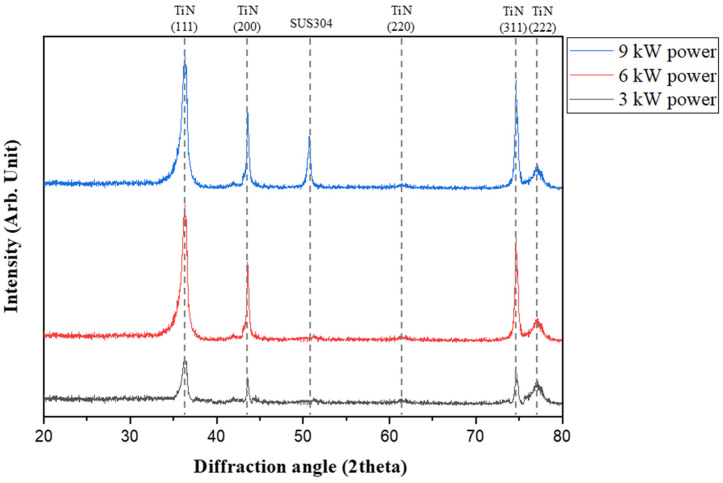
HR-XRD patterns of TiN coatings by X-ray power.

**Figure 3:**
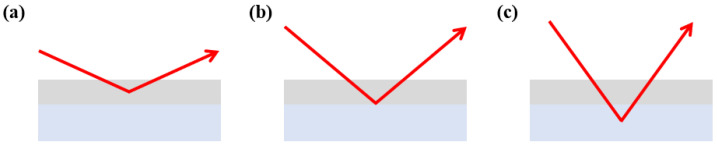
Difference in penetration depth and analysis by change in X-ray power: (**a**) 3 kW power; (**b**) 6 kW power; (**c**) 9 kW power.

Figure 2 shows the XRD peaks for the (111) plane, examining the variations in ψ according to stress changes in stress mode relative to the X-ray power. The gradient of diagram, representing the variation in internal stress according to each power, was calculated. The results showed that the gradient remained constant, indicating that even if the high power of the X-ray affected the substrate, there was no issue in evaluating the internal stress, as only the peak of the TiN coating was analyzed. As shown in Figure 3, the X-ray must penetrate into the substrate to accurately assess the internal stress of the entire coating layer.

**Figure 4:**
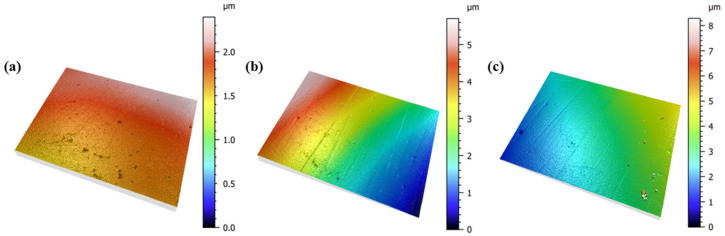
Surface roughness of TiN coatings as determined by confocal microscope: (**a**) 0.01 Sa; (**b**) 0.02 Sa; (**c**) 0.03 Sa.

To evaluate internal stress based on surface roughness, specimens were prepared with the same coating thickness, and the surface roughness was adjusted through polishing after the deposition process. The surface roughness was adjusted to 0.01 Sa, 0.02 Sa, and 0.03 Sa, and the images showing the surface roughness are shown in Figure 4. The X-ray power was set to 9 kW to cover the entire coating layer, and the internal stress was analyzed in a stress mode, focusing on the (111) plane. The gradient of diagram was found to be constant for all three specimens, confirming that the method is valid for coating specimens with a surface roughness of 0.03 Sa or less. In general, considering that the surface roughness of titanium-based nitride coatings deposited by sputtering is 0.02 Sa or less, the test method for internal stress using HR-XRD is suitable.

**Figure 5:**
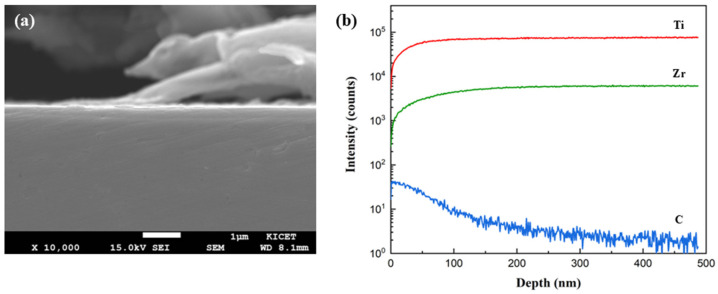
(**a**) Cross-sectional SEM images of carbon-doped TiZrN coating; (**b**) ToF-SIMS depth profiles of carbon-doped TiZrN coating.

Figure 5 shows a cross-sectional SEM image of a carbon-doped TiZrN coating, with ToF-SIMS data showing the carbon concentration with depth. The ToF-SIMS data showed that the carbon diffused down to about 0.4 μm, and so the thickness of the coating was adjusted to 0.5 μm, 1 μm, and 2 μm. If the laser carburization affects the interface or substrate rather than the coating, damage such as delamination or degradation can occur, reducing the durability of the coating. In this study, since the minimum thickness of the coating is 0.5 μm, it is not expected to affect the interface or substrate, and no damage to the coating was observed in the cross-sectional SEM images.

**Figure 6:**
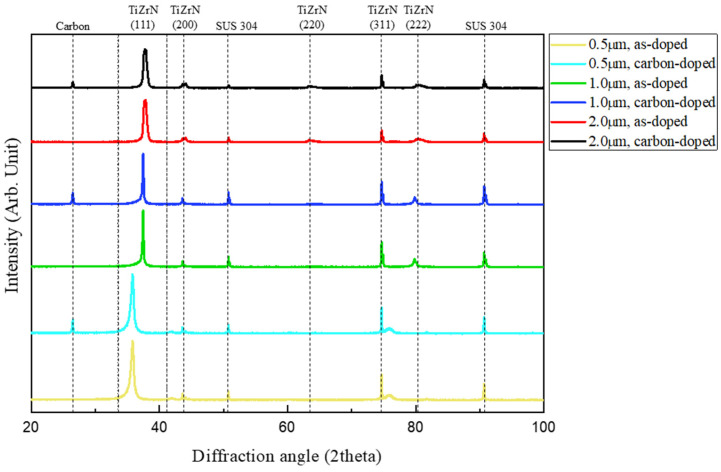
HR-XRD patterns of as-doped and carbon-doped TiZrN coatings by coating thickness.

To analyze the effect of carbon doping on the ratio of the carbon gradient layer, the crystal structure was examined using HR-XRD. Figure 6 shows the HR-XRD peaks corresponding to the ratio of the carbon gradient layer before and after carbon doping. In previous research, it was confirmed through TEM image analysis that the columnar grain structure was formed on the TiZrN (111) plane, which was the preferred growth orientation, and the peak of TiZrN (111) plane with strong peak intensity was also confirmed in this research [16,17]. After laser carburization, carbon doping was confirmed by the formation of carbon peaks, which tended to be more pronounced as the ratio of the carbon gradient layer increased.

Rietveld refinement was performed based on HR-XRD to calculate the change in the lattice constant and is shown in Table 2. For all coating thicknesses, the lattice constant increased with carbon doping. The lattice constant increased from 4.12 Å to 4.38 Å, and the increase in lattice constant was more significant as the ratio of the carbon gradient layer increased. This appears to be caused by lattice distortion, as carbon atoms, which have a larger atomic radius than nitrogen, enter the TiN lattice in substitutional and interstitial forms [18].

The sin^2^ψ method was utilized to analyze the effect of lattice distortion, caused by carbon doping, on the internal stress of the coating. Table 3 and Table 4 show the parameters required for calculating internal stress before and after carbon doping according to the coating thickness. After carbon doping, internal stress increased in all coating thicknesses, exhibiting negative values indicative of compressive stress. The internal stress increased by 33% from −1263 MPa to −1687 MPa, and the internal stress increased the most at 0.5 μm, where the ratio of the carbon gradient layer was the largest.

**Figure 7:**
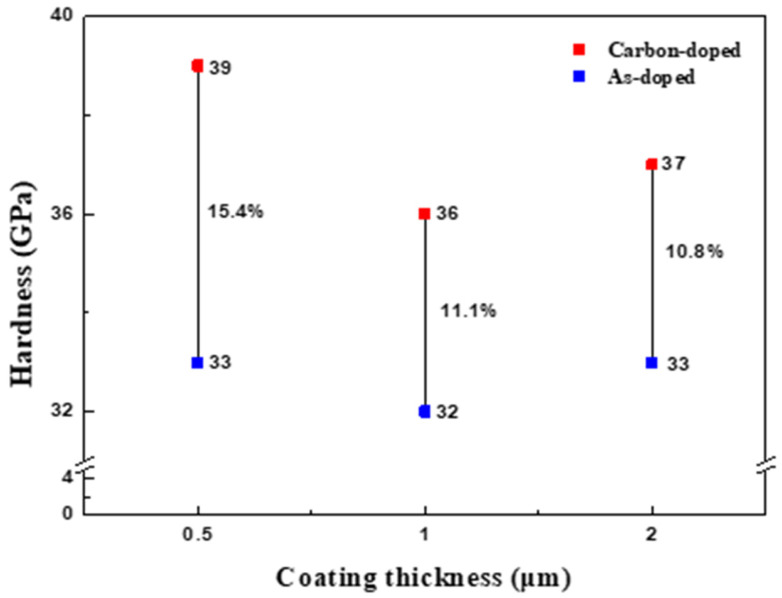
Hardness of as-doped and carbon-doped TiZrN coatings by coating thickness.

Figure 7 shows the changes in hardness before and after carbon doping depending on the ratio of the carbon gradient layer measured by a nanoindenter. For all coating thicknesses, the hardness before carbon doping was similar, but as the ratio of the carbon gradient layer increased, the difference in hardness changes emerged. The hardness increases at 0.5 μm, 1 μm, and 2 μm thicknesses were 15.4%, 11.1%, and 10.8%, respectively. It was confirmed that the hardness was improved the most from 33 GPa to 39 GPa at the thickness of 0.5 μm, where the ratio of the carbon gradient layer was the highest. This improvement can likely be attributed to the enhanced mechanical properties resulting from increased internal stress. Previous research has been conducted on titanium nitride coatings with carbon, and the results of this research show similar or better property improvements compared to previous research [19,20,21].

## 4. Conclusions

The test method for internal stress of titanium-based nitride was optimized using the sin^2^ψ method with HR-XRD. Through the test method, the variations in mechanical properties by the ratio of the carbon gradient layer were investigated in terms of internal stress. TiN coatings were deposited on SUS 304 as the substrate with the same coating thickness of 1 μm, and the internal stress according to the penetration depth and surface roughness was analyzed in the stress mode of HR-XRD for the (111) plane, which is the preferred growth orientation. The penetration depth was adjusted by varying the X-ray power of HR-XRD. The gradient of diagram for internal stress remained constant regardless of the penetration depth, and it was attributed to the analysis of internal stress focusing on the preferred growth orientation of the coating, excluding the influence of the substrate. In addition, the test was conducted on specimens with different surface roughness values (0.01 Sa, 0.02 Sa, 0.03 Sa) and it was confirmed that the difference in internal stress was insignificant. As such, this test method was valid at a surface roughness of 0.03 Sa or less. Subsequently, the test method for internal stress was applied to investigate the variations in the mechanical properties of carbon-doped TiZrN coatings based on the ratio of the carbon gradient layer. To adjust the ratio of the carbon gradient layer within the coating, the coating thicknesses were set to 0.5 μm, 1 μm, and 2 μm. Crystal structure analysis confirmed the formation of the peak of the (111) plane, which is the preferred growth orientation of TiZrN, along with the carbon peak after carbon doping. In addition, the lattice constant changes were calculated using Rietveld refinement, and as the ratio of the carbon gradient layer increased, the lattice constant increased from 4.12 Å to 4.38 Å, indicating lattice distortion. Accordingly, the variation in internal stress was confirmed, and the internal stress increased the most from −1263 MPa to −1687 MPa at 0.5 μm, where the ratio of the carbon gradient layer was the highest. As a result of analyzing the mechanical properties of internal stress variations, the hardness increased from 33 GPa to 39 GPa as the ratio of the carbon gradient layer changed. The results showed that mechanical properties improved most significantly at a coating thickness of 0.5 μm, which had the highest ratio of carbon gradient layer, while similar levels of improvement were observed at a thickness of 1 μm and 2 μm.

## Figures and Tables

**Table 1:** Deposition conditions of TiN and TiZrN coating on SUS 304.

Coating thickness (μm)	0.5	1	2
Deposition time (h)	1	2	4
Base pressure (Torr)	1.0 × 10^−5^		
Working pressure (Torr)	1.0 × 10^−2^		
Rotational velocity of substrate (rpm)	15		
RF power (W)	200		
Ar:N_2_ gas ratio	3:1		
Deposition temperature (K)	723		

**Table 2:** Lattice constants with different coating thickness.

	0.5 μm		1 μm		2 μm	
	As-Doped	Carbon-Doped	As-Doped	Carbon-Doped	As-Doped	Carbon-Doped
Lattice constant (Å)	4.12	4.38	4.13	4.34	4.15	4.33
Variation (Å)	0.26		0.21		0.18	

**Table 3:** Various factors in calculation of stress constant.

	0.5 μm		1 μm		2 μm	
	As-Doped	Carbon-Doped	As-Doped	Carbon-Doped	As-Doped	Carbon-Doped
E_IT_ (GPa)	314	338	315	335	315	332
E (GPa)	416	461	418	455	419	450
ν	0.201	0.202	0.201	0.202	0.201	0.202
2θ_0_ (°)	37	37	37	37	37	37
K	−9033	−10,003	−9077	−9873	−9099	−9764

**Table 4:** Stress constant and gradient of diagram for calculation of internal stress.

	0.5 μm		1 μm		2 μm	
	As-Doped	Carbon-Doped	As-Doped	Carbon-Doped	As-Doped	Carbon-Doped
Stress constant (K)	−9033	−10,003	−9077	−9873	−9099	−9764
Gradient of diagram (M)	0.140	0.169	0.141	0.161	0.141	0.168
Internal stress (MPa)	−1263	−1687	−1278	−1592	−1284	−1645
Variation (%)	33		25		28	

## Data Availability

Data are contained within the article.

## References

[1] Venkatesan G., Jithin P.R., Rajan T.V., Pitchan M.K., Bhowmik S., Rane R., Mukherjee S. (2018). Effect of titanium nitride coating for improvement of fire resistivity of polymer composites for aerospace application. Proc. Inst. Mech. Eng. Part G J. Aerosp. Eng..

[2] Ezazi M.A., Quazi M.M., Zalnezhad E., Sarhan A.A. (2014). Enhancing the tribo-mechanical properties of aerospace AL7075-T6 by magnetron-sputtered Ti/TiN, Cr/CrN & TiCr/TiCrN thin film ceramic coatings. Ceram. Int..

[3] Wróblewski P., Rogólski R. (2021). Experimental analysis of the influence of the application of TiN, TiAlN, CrN and DLC1 coatings on the friction losses in an aviation internal combustion engine intended for the propulsion of ultralight aircraft. Materials.

[4] Kenzhegulov A., Mamaeva A., Panichkin A., Alibekov Z., Kshibekova B., Bakhytuly N., Wieleba W. (2022). Comparative study of tribological and corrosion characteristics of TiCN, TiCrCN, and TiZrCN coatings. Coatings.

[5] Tillmann W., Grisales D., Stangier D., Thomann C.A., Debus J., Nienhaus A., Apel D. (2021). Residual stresses and tribomechanical behaviour of TiAlN and TiAlCN monolayer and multilayer coatings by DCMS and HiPIMS. Surf. Coat. Technol..

[6] Knotek O., Münz W.D., Leyendecker T. (1987). Industrial deposition of binary, ternary, and quaternary nitrides of titanium, zirconium, and aluminum. J. Vac. Sci. Technol. A Vac. Surf. Film..

[7] Riyadi T.W.B., Setiadhi D., Anggono A.D., Siswanto W.A., Al-Kayiem H.H. (2021). Analysis of mechanical and thermal stresses due to TiN coating of Fe substrate by physical vapor deposition. Forces Mech..

[8] Sarmast A., Schubnell J., Preußner J., Hinterstein M., Carl E. (2023). Residual stress analysis in industrial parts: A comprehensive comparison of XRD methods. J. Mater. Sci..

[9] Meng Z., Tian C., Li P. (2018). The relationship between tensile strain and residual stress of high strength dual phase steel sheet. MATEC Web of Conferences.

[10] Arunkumar P., Panda P., Sribalaji M., Ramaseshan R., Keshri A.K., Babu K.S. (2017). Enhancing the oxygen ionic conductivity of (111) oriented Ce_0.80_Sm_0.20_O_2-δ_ thin film through strain engineering. Electrochim. Acta.

[11] Dobrzański L.A., Dobrzański L.B., Dobrzańska-Danikiewicz A.D. (2020). Manufacturing technologies thick-layer coatings on various substrates and manufacturing gradient materials using powders of metals, their alloys and ceramics. J. Achiev. Mater. Manuf. Eng..

[12] Dobrzański L.A., Żukowska L.W., Mikuła J., Gołombek K., Pakuła D., Pancielejko M. (2008). Structure and mechanical properties of gradient PVD coatings. J. Mater. Process. Technol..

[13] Karimoto T., Nishimoto A. (2019). Simultaneous boronizing and carburizing of titanium via spark plasma sintering. Mater. Trans..

[14] Matsubara M., Bellotti E. (2017). A first-principles study of carbon-related energy levels in GaN. I. Complexes formed by substitutional/interstitial carbons and gallium/nitrogen vacancies. J. Appl. Phys..

[15] Chen T., Foo C., Tsang S.C.E. (2021). Interstitial and substitutional light elements in transition metals for heterogeneous catalysis. Chem. Sci..

[16] Kim S., Kim T., Hong E., Jo I., Kim J., Lee H. (2021). Phase formation and wear resistance of carbon-doped TiZrN nanocomposite coatings by laser carburization. Metals.

[17] Hong E., Jeon S., Choi Y., Yoo H., Kwon S.H., Lee H. (2018). Lattice distortion and residual stress of a carbon-doped TiZrN coating. Int. J. Appl. Ceram. Technol..

[18] Hong E., Kim T., Kim S., Lee S.H., Lee H. (2021). Carbon depth profile and internal stress by thermal energy variation in carbon-doped TiZrN coating. J. Am. Ceram. Soc..

[19] Braic V., Braic M., Balaceanu M., Vladescu A., Zoita C.N., Titorencu I., Jinga V. (2011). (Zr, Ti) CN coatings as potential candidates for biomedical applications. Surf. Coat. Technol..

[20] Braic M., Balaceanu M., Vladescu A., Zoita C.N., Braic V. (2011). Study of (Zr, Ti) CN,(Zr, Hf) CN and (Zr, Nb) CN films prepared by reactive magnetron sputtering. Thin Solid Film..

[21] Liang L., Wei B., Wang D., Fang W., Chen L., Wang Y. (2022). Densification, microstructures, and mechanical properties of (Zr, Ti)(C, N) ceramics fabricated by spark plasma sintering. J. Eur. Ceram. Soc..

